# Personalized follow-up of circulating DNA in resected stage III/IV melanoma: PERCIMEL multicentric prospective study protocol

**DOI:** 10.1186/s12885-023-11029-4

**Published:** 2023-06-16

**Authors:** Lionnel Geoffrois, Alexandre Harlé, Nassim Sahki, Aleksandra Sikanja, Florence Granel-Brocard, Alice Hervieu, Laurent Mortier, Géraldine Jeudy, Catherine Michel, Charlée Nardin, Cécile Huin-Schohn, Jean-Louis Merlin

**Affiliations:** 1 Medical Oncology Department, Institut de Cancérologie de Lorraine, Vandoeuvre Les Nancy, France; 2 Biopathology Department, Institut de Cancérologie de Lorraine, CNRS UMR7039 CRAN Université de Lorraine, Vandoeuvre Les Nancy, France; 3 Methodology Biostatistics Unit, Institut de Cancérologie de Lorraine, Vandoeuvre Les Nancy, France; 4 Clinical Research Department, Institut de Cancérologie de Lorraine, Vandoeuvre Les Nancy, France; 5 Dermatology Department, CHRU Nancy, Vandoeuvre Les Nancy, France; 6 Medical Oncology Department, Centre Georges François Leclerc, Dijon, France; 7 Dermatology Department CHRU Lille, Inserm U1189, Université de Lille, Lille, France; 8 Dermatology Department, CHU Dijon, Dijon, France; 9 Dermatology Department, GHR Mulhouse Sud Alsace, Mulhouse, France; 10 Dermatology Department CHU Besançon, Inserm 1098 RIGHT Université Franche Comté, Besançon, France

**Keywords:** Circulating tumor DNA, Melanoma, Adjuvant therapy, Immunotherapy, Kinase inhibitors

## Abstract

**Background:**

With more than 15,000 new cases /year in France and 2,000 deaths, cutaneous melanoma represents approximately 4% of incidental cancers and 1.2% of cancer related deaths. In locally advanced (stage III) or resectable metastatic (stage IV) melanomas, medical adjuvant treatment is proposed and recent advances had shown the benefit of anti-PD1/PDL1 and anti-CTLA4 immunotherapy as well as anti-BRAF and anti-MEK targeted therapy in BRAF V600 mutated tumors. However, the recurence rate at one year is approximately 30% and justify extensive research of predictive biomarkers. If in metastatic disease, the follow-up of circulating tumor DNA (ctDNA) has been demonstrated, its interest in adjuvant setting remains to be precised, especially because of a lower detection rate. Further, the definition of a molecular response could prove useful to personalized treatment.

**Methods:**

PERCIMEL is an open prospective multicentric study executed through collaboration of the Institut de Cancérologie de Lorraine (non-profit comprehensive cancer center) and 6 French university and community hospitals. A total of 165 patients with resected stage III and IV melanoma, eligible to adjuvant imunotherapy or anti-BRAF/MEK kinase inhibitors will be included. The primary endpoint is the presence of ctDNA, 2 to 3 weeks after surgery, defined as mutated ctDNA copy number calculated as the allelic fraction of a clonal mutation relative to total ctDNA. Secondary endpoints are recurrence-free survival, distant metastasis-free survival and specific survival. We will follow ctDNA along treatment, quantitatively through ctDNA mutated copy number variation, qualitatively through the presence of cfDNA and its clonal evolution. Relative and absolute variations of ctDNA during follow-up will be also analyzed.

PERCIMEL study aims at provide scientific evidence that ctDNA quantitative and qualitative variations can be used to predict the recurrence of patients with melanoma treated with adjuvant immunotherapy or kinase inhibitors, thus defining the notion of molecular recurrence.

## Background

With an estimated 15,500 new cases in 2018 in metropolitan France (7,900 men and 7,600 women), and 1,880 deaths (1,040 men and 840 women), cutaneous melanoma accounts for around 4% of all incident cancers, and 1.2% of all cancer-related deaths among both sexes. It is one of the forms of cancer that has seen its incidence and mortality rise significantly in the last 40 years (data from the National Cancer Institute, www.e-cancer.fr). At locally advanced or metastatic stages, relative survival at 5 years is around 60% in patients with loco-regional cancer, and 15% in those with metastasis. At earlier stages of the disease, relative 5-year survival is above 90%, but although patients can be potentially cured by surgery, 13% will go on to develop loco-regional or metastatic disease within 2 years.

Standard treatment for locally advanced melanoma that is amenable to surgery (stage III disease) is surgical resection, with lymph node dissection in case of macroscopic lymph node involvement, or sentinel node biopsy in the absence of detectable macroscopic lymph node involvement. For metastatic melanoma (stage IV), metastasis amenable to complete surgical removal without residual disease should undergo surgery. In these cases, adjuvant medical therapy is proposed.

Since the advent of the first adjuvant treatments based on interferon α2b, the therapeutic arsenal has increased considerably, with immunotherapies such anti-PD1 [1, 2], and targeted therapies using the tyrosine kinase BRAF and MEK inhibitors for patients harbouring activating mutations of the BRAF gene [3].

Recently published analyses reporting 3 to 5-year survival have confirmed the value of adjuvant treatment for operable stage III and IV melanoma. Accordingly, in the randomized, phase III KEYNOTE-054 study, which investigated the anti-PD1 monoclonal antibody pembrolizumab versus placebo in resected high-risk stage III melanoma, the results showed a significant prolongation of recurrence-free survival (RFS) at 3 years (63.7% vs 44.1%, hazard ratio (HR), 0.56; 95% confidence interval (CI), 0.47 to 0.68) [4]. Similar findings were also reported from the randomized, phase III Checkmate 238 study, which tested the anti-PD1 monoclonal antibody nivolumab versus immunotherapy with the anti-CTLA4 monoclonal antibody ipilimumab in patients with resected stage IIIB–C or stage IV melanoma, and found a sustained RFS benefit in favour of nivolumab at 4 years (51.7% versus 41.2% with ipilimumab (HR, 0.71, 95% CI 0.60–0.86) [5]. In addition, in the randomized, phase III Combi-AD trial, evaluating the association of the BRAF inhibitor dabrafenib and the MEK inhibitor trametinib versus placebo in patients with BRAFV600 mutant melanoma, the results showed a significant improvement in RFS at 5 years, with 52% RFS in the dabrafenib plus trametinib arm versus 36% in the control group (HR, 0.51; 95% CI, 0.42 to 0.61) [6].

In a recent published paper, [7] the authors presented the results of a neoadjuvant and adjuvant or adjuvant only Pembroluzumab in locally advances melanoma. They included 313 patients, 154 in the neoadjuvant-adjuvant arm and 159 in the adjuvant only arm. All patients had clinically detectable measurable disease stage IIIB to IIID melanoma or oligometastatic resectable stage IV. Primary objective was EFS (Event Free Survival). With a median follow up of 14,7 months they demonstrated significant benefit for the neoadjuvant – adjuvant arm, with an EFS of 72% at 2 years versus 49% in the adjuvant arm with a *p* value at 0.004. This new strategy will be considered very soon as a standard of care, and needs to be integrated in our study, an amendment is in preparation.

However, the rate of relapse at one year after the end of treatment is around 30%, underlining the need to find predictive markers of early relapse [6].

In this context, analysis of circulating tumor DNA (ctDNA) is of relevance. The presence of nucleic acid in the blood was first described in 1948, but it has only recently been discovered that tumours are capable of actively and passively releasing their DNA into body fluids (for review, see [8]). Technological progress, notably in molecular biology, now makes it possible to detect ctDNA and to envisage clinical applications using ctDNA detection in the field of medical oncology. The detection of ctDNA, *i.e.* genetic material released by the tumour during necrosis, apoptosis or other active cell phenomena, could be used for the early detection of cancer, for theranostic applications, for follow-up of patients, to evaluate the quality of surgery or for the early detection of relapse [8]. Indeed, it has been shown that the concentration of ctDNA is correlated to tumour burden and consequently, is higher in patients with metastasis.

In the setting of melanoma, the majority of studies have been performed in metastatic, inoperable disease [9–17]), where ctDNA was more easily detectable, and frequently observed (70–90%).

These studies were performed in patients with non-resectable, stage III or metastatic (stage IV) melanoma, treated with BRAF inhibitors, with or without MEK inhibitors. The concentration of ctDNA before, and 4 weeks after treatment was shown to be a prognostic factor for survival [16]. Prior to the advent of adjuvant therapies, studies also showed the utility of ctDNA in operable advanced melanoma: ctDNA at 12 weeks after surgery seemed to be of use for predicting survival in patients with operable stage II or III melanoma [18], and also in patients with stage III disease, independently of the substage (a/b/c/d), when ctDNA was measured pre-operatively [19, 20]) or post-operatively [20].

A study of non-resectable advanced melanoma treated by immunotherapy or targeted therapy, with or without chemotherapy, showed that variations in ctDNA during follow-up were an indicator of treatment efficacy, suggesting that ctDNA could be a better reflection of tumoral heterogeneity than tissue biopsy [21].

In patients who undergo surgery, the situation is different, and notably, ctDNA can only be detected in a much smaller percentage of cases (20–40%). Few studies were performed before the advent of adjuvant treatment with immunotherapy or BRAF and MEK inhibitors. For example, the study by Tan et al. [20] in patients with operable, stage III melanoma found that the detection of ctDNA at baseline and after treatment was associated with shorter RFS and inferior distant metastasis-free survival (DMFS). In the same way, other studies [18, 19] performed respectively in 161 stage II/III high-risk melanoma patients and 174 patients with stage III melanoma undergoing complete lymph node dissection, found that the detection of ctDNA at baseline was associated with a higher rate of LN involvement, high lactate dehydrogenase (LDH) levels and worse melanoma-specific survival [19], and the presence of ctDNA post-operatively was associated with shorter metastasis-free and overall survival.

These studies also highlight the value of ctDNA for the prediction of survival in patients with operable stage II and III melanoma [18], and in stage III, regardless of substage (a/b/c/d) when ctDNA is measured at baseline [19, 20] or post-operatively [20]. More recently, the study by Gouda et al. [22] in 80 patients with BRAFV600E mutated melanoma of stage ≤ III, detection of ctDNA after surgery was associated with a higher likelihood of melanoma recurrence and shorter disease-free and overall survival. Furthermore, analyses of ctDNA from patients included in the Checkmate 915 study found that the presence of ctDNA predicted early relapse and inferior progression-free and metastasis-free survival, in patients with resected stage IIIB-D/IV melanoma receiving adjuvant nivolumab with or without ipilimumab [23]).

In these studies, which were almost all performed before the introduction onto the market of kinase inhibitors and immunotherapy in the adjuvant setting, none investigated normalized follow-up of ctDNA during treatment, or the link between the course of ctDNA and therapeutic response.

In the current era of adjuvant therapy, one of the aims of the PERCIMEL study is to determine whether the monitoring of ctDNA makes it possible to predict relapse, thereby defining the concept of molecular relapse.

## Objectives

The primary objective of PERCIMEL study is to evaluate the predictive value of the presence of ctDNA post-surgery on recurrence-free survival at 24 months in patients undergoing surgery for cutaneous melanoma and treated with immunotherapy or targeted therapy with BRAF and MEK inhibitors, in an adjuvant setting.

The secondary objectives are:

- To evaluate the predictive value of baseline (prior to surgery) ctDNA on survival (recurrence-free survival, distant metastasis-free survival and specific survival).
- To analyse the quantitative and clonal course of ctDNA during 24 months of follow-up, or at relapse, to predict survival
- To describe the clinical characteristics and the quantitative course of ctDNA during follow-up in patients with no detectable ctDNA at pré and/or post-surgery
- To determine the predictive value of ctDNA course in subgroups of patients according to clinical and tumour characteristics at inclusion.

## Method/design

The PERCIMEL study is an Interventional multicenter cohort study with minimal risks and constraints.

**Fig. 1:**
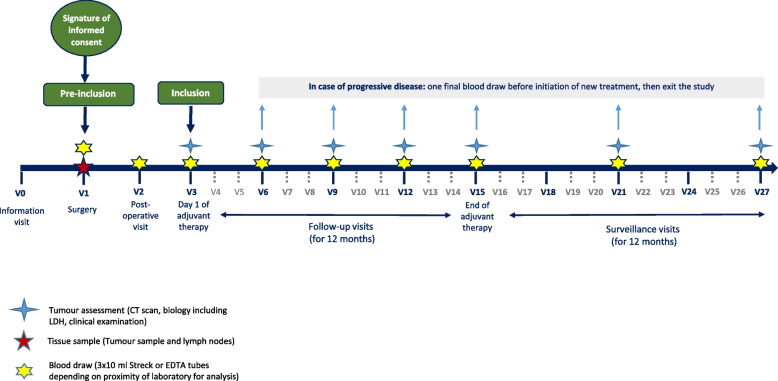
Percimel protocol scheduling

Figure 1 summarizes the study scheduling. The study is registered on ClinicalTrials.gov (NCT04866680), and French Competent Authorithy (ID-RCB: 022-A01904-39).

### Study population

The PERCIMEL study is proposed to patients diagnosed with histologically-proven cutaneous melanoma, locally advanced, resectable, with complete lymph node dissection upfront in patients presenting with macroscopic lymph node involvement, or with positive sentinel lymph node biopsy (microscopic lesions) or surgery of distant metastasis, followed by adjuvant therapy with immunotherapy or targeted therapy with BRAF and MEK inhibitors, according to currently approved indications.

### Inclusion criteria

- Patient aged 18 years or older
- WHO performance status 0–2
- Patient with histologically-proven cutaneous melanoma, locally advanced (stage III) or metastatic and amenable to complete resection (stage IV)
- Naïve of all treatments (except for initial biopsy for diagnostic purposes)
- Patient with an indication for adjuvant therapy with BRAF and MEK inhibitors or immunotherapy with anti-PD1 monoclonal antibodies, according to currently approved indications
- Biological parameters compatible with the proposed treatment
- Patient affiliated to a social security regimen, or beneficiary thereof
- Signature of informed consent form

### Non-inclusion criteria

- Patients with mucous melanoma or choroidal (uveal) melanoma
- Patient presenting with a synchronous tumour, or having been treated in the 3 years prior to pre-inclusion (except for cervical carcinoma in situ, or resected cutaneous carcinoma)
- Patients with a contra-indication to blood draw of 30 ml
- Patients with a contra-indication to surgery
- Patients with a contra-indication to the proposed adjuvant therapy
- Pregnant or breastfeeding women
- Patients under any form of judicial or legal protection

### Sample size calculation

Our hypothesis is that 25% of patients will have detectable ctDNA post-operatively, i.e. within 2 to 3 weeks after surgery [20].

Recurrence-free survival at 24 months in this population, based on key studies of adjuvant therapies, can be estimated at 70% (upper limit, to ensure a good statistical power) [1, 2, 24].

Based on an inclusion period of 24 months, an alpha risk of 5% and statistical power of 90%, a total of 124 patients would be required to achieve a hazard ratio of 2.5 [18, 20]. By considering 10% of patients lost to follow-up at 24 months or not analysable, then a total of 136 patients are necessary.

It is expected that almost all patients with stage IIIc/IIId/IV melanoma who are pre-included will subsequently be definitively included. Conversely, we estimate that around 70% of patients with stage IIIa-IIIb melanoma who are pre-included will have negative sentinel lymph nodes and therefore, will not be eligible for definitive inclusion. In view of the active pool of patients, we expect that stage IIIa-IIIb patients will account for 25% of all pre-included patients. Thus, to achieve definitive inclusion of 136 patients, we estimate that 165 patients will need to be pre-included.

### Statistical analysis

The main objective is to evaluate the predictive value of the presence of ctDNA post-operatively on RFS. The RFS will be described by the Kaplan–Meier method, and compared according to the presence or absence of ctDNA post-surgery using the log rank test. A multivariate Cox regression model will be used to calculate the hazard ratio and 95% confidence interval (CI). As this is a cohort study, the results will be adjusted for baseline patient characteristics and the type of adjuvant therapy. To this end, bivariate analyses will be performed to identify variables related to the RFS. Variables yielding a *p*-value < 0.10, and the presence of ctDNA post-surgery will be included in the multivariate model. Results will be presented as hazard ratios with 95% CIs.

The same analysis will be performed for ctDNA presence pre-surgery, for the number of copies of mutant DNA per ml of plasma pre- and post-surgery, and for absolute and relative variations of the quantity of mutated DNA between pre- and post-surgery.

The impact of ctDNA evolution during follow-up on RFS will be evaluated using time-dependent survival models, by considering mutated DNA copy number as a time-dependent variable in the Cox model. This analysis will be adjusted for baseline patient characteristics and for the type of adjuvant therapy to assess whether the course of ctDNA during follow-up is an independent prognostic factor for survival.

To determine whether the predictive value of the course of ctDNA varies according to clinical and tumour characteristics at baseline, interaction tests will be performed.

Distant metastasis-free survival will be analysed using the same approach as for RFS. Melanoma-specific survival will also be analysed using the same approach, but considering death unrelated to the disease as a competing risk. Consequently, specific survival will be described as cumulative incidence according to the Kalbfleisch-Prentice method, and compared using Gray’s test. Bi- and multivariate analysis will be performed using the Fine and Gray subdistribution model.

Baseline characteristics of the study population will be compared between patients with and patients without detectable ctDNA pre- and/or post-operatively. Qualitative variables will be compared using the chi square or Fisher’s exact test, and quantitative variables using the Student t or Mann–Whitney U test, according to whether the distribution of the variable is normal or not. Normality will be tested using the Shapiro–Wilk test. A multivariate logistic regression model will be fit to determine the characteristics that best distinguish the two populations. The discriminant power of the model will be determined using the area under the receiving operating characteristic (ROC) curve. In patients with no detectable ctDNA either pre- or post-operatively, the course of quantification of ctDNA will be described by repeated measure ANOVA using the mixed linear model, to take account of the correlation between measures in a same patient. Time will be considered as a fixed effect, and the patient as a random effect. The analysis will be performed in patients who have progressive disease, and those who do not.

The cut-off timepoint for this analysis will be the date of progression or end of follow-up. The values analysed will be number of copies of mutant DNA, taking account of all values obtained since inclusion.

All statistical analyses will be performed using SAS version 9.4 (SAS Institute Inc., Cary, NC, USA). A *p*-value < 0.05 will be considered statistically significant.

## Study procedures

### Practical implementation of the study

All study visits will be scheduled to coincide with the standard follow-up appointments. Each patient will have a maximum of 9 blood draws.

### Study information and pre-inclusion visit

All patients meeting the following criteria will be eligible for pre-inclusion in the PERCIMEL study before scheduled surgery, i.e.:

- Patient presenting with histologically confirmed cutaneous melanoma
- Stage III, and staging has confirmed the absence of distant metastasis,
  1. with no detectable macroscopic lymph node involvement, and for whom there is an indication for completion lymph node biopsy and investigation of sentinel lymph node (Breslow > 0.8 mm, or regardless of Breslow index in the presence of ulceration)
  2. with macroscopically detectable lymph node involvement (either synchronously at the time of discovery of the melanoma, or metachronously) and with an indication for complete lymph node dissection
- Stage IV, and staging has confirmed the presence of distant metastasis, stage IV, amenable to complete resection, R0.

Information about the PERCIMEL study (and the associated biological collection) will be given to eligible patients by the clinicians in the participating centre during a medical consultation including:

- Complete clinical examination.
- Assessment of general state (WHO score) and body weight
- Collection of personal history of melanoma
- Collection of the results of staging examinations (radiological data): echography, abdominal-thoracic-pelvic CT scan, PET scan or brain CT scan
- Collection of pathology results with histological confirmation of cutaneous melanoma (from initial biopsy for diagnostic purposes)
- Collection of concomitant treatment (any treatment usually taken by the patient at the time of pre-inclusion)
- Check for eligibility criteria.

After sufficient time for reflection, and after obtaining the patient’s written informed consent, the patient will be pre-included.

### Surgery

Surgery will be performed according to standard practices in line with the recommendations for management of melanoma, and in general, within 28 days after the pre-inclusion visit.

For all pre-included patients:

- A blood draw of 30 ml for ctDNA analysis will be performed on the evening before, or on the day of surgery, before the start of surgery.
- Tissue samples (operative tumour samples and lymph nodes) will be retrieved and handled according to standard practices.

### Post-operative visit

For all pre-included patients, the post-operative visit will be performed within 2 to 3 weeks after surgery, and will consist in:

- Recording peri-operative complications (infected lymphocele, lymphoedema, scar disunion…) of grade > 2 or of any grade (according to the CTCAE classification version 5) if the impact on further management is significant
- A blood draw of 30 ml on the day of the post-operative visit for the purposes of the study.

### Inclusion visit

Patients will be definitively included if histology confirms:

- Positive lymph nodes for patients with stage III melanoma
- The presence of metastasis for patients with stage IV melanoma

For all patients included, adjuvant therapy must be initiated within 12 weeks after surgery. Adjuvant therapy by immunotherapy or targeted therapy with BRAF and MEK inhibitors will be planned for 12 months, as per standard recommendations.

The inclusion visit will consist in:

- A clinical examination of the skin, scalp and lymph node basins, to note any signs and symptoms
- Assessment of general state (WHO score) and body weight
- Biology, including complete blood count, hemostasis, blood gases, plasma creatinine, liver function tests, and any other biological parameter required for the implementation of adjuvant medical therapy.
- Pathology results of the operative sample and/or sentinel lymph nodes
- Recording of any adverse events grade > 2 or adverse events of any grade (according to the CTCAE classification version 5) if the impact on medical therapy or further management is significant
- Recording of concomitant treatments (if there are any changes compared to those recorded at the pre-inclusion visit)
- A blood draw of 30 ml, performed on the day of the consultation where adjuvant therapy is initiated, and before the administration of adjuvant therapy.
- Patients who consented to pre-inclusion but who are not definitively included (negative sentinel lymph node, or metastasis not confirmed by lymph node dissection) will exit the study at this point and will be managed as per standard recommendations.

### Follow-up visits during adjuvant therapy (up to 12 months)

All follow-up visits will be scheduled to coincide with the standard follow-up appointments, i.e. every 3 to 4 months ± 2 weeks, in order to avoid the patient having to attend visits solely for the purposes of the study.

Each follow-up visit will include:

- Complete clinical examination
- Assessment of general state (WHO score) and body weight
- Biology, including complete blood count, hemostasis, blood gases, plasma creatinine, liver function tests, and any other biological parameter required for the implementation of adjuvant medical therapy.
- Recording of any adverse events grade > 2 or adverse events of any grade (according to the CTCAE classification version 5) if the impact on medical therapy or further management is significant
- Recording of concomitant treatments (if there are any changes compared to those recorded at the pre-inclusion visit)
- Tumour assessment
- A blood draw of 30 ml for ctDNA analysis, performed on the day of each follow-up visit

In case of relapse during the 12 months of follow-up, the patient will have one final 30 ml blood draw and will then exit the study.

### Follow-up visits (from 12 to 24 months)

All follow-up visits will be scheduled to coincide with the standard follow-up appointments, in order to avoid the patient having to attend visits solely for the purposes of the study. Follow-up visits will be planned according to current guidelines. For the purposes of the study, a follow-up visit will be planned every 6 months ± 2 weeks.

Appropriate, systematic radiological examination (CT or PET scan) is recommended every 6 months during the 12 months of follow-up.

Each follow-up visit will consist in:

- Complete clinical examination
- Assessment of general state (WHO score) and body weight
- Recording of any adverse events grade > 2 or adverse events of any grade (according to the CTCAE classification version 5) if the impact on medical therapy or further management is significant
- Recording of concomitant treatments (if there are any changes compared to those recorded at the pre-inclusion visit)
- Tumour assessment
- A blood draw of 30 ml for ctDNA analysis, performed on the day of each follow-up visit

After the 12 months of follow-up, the patient will have one final blood draw and will then exit the study.

In case of relapse during the 12 months of surveillance, the patient will have one final blood draw and will then exit the study.

### Tumour assessment

Tumour assessment will include:

- Clinical examination
- CT or PET scan or brain CT scan
- Biology, including measurement of LDH

Tumour assessment will be scheduled every 3 to 4 months ± 2 weeks during adjuvant therapy and every 6 months ± 2 weeks during follow-up, according to local practices in each centre.

Progressive disease is defined as the appearance of a new clinical or radiological lesion.

In case of progressive disease, the visit will consist in:

- A clinical examination of the skin, scalp and lymph node basins, to note any signs and symptoms
- Assessment of general state (WHO score) and body weight
- Biology, including complete blood count, hemostasis, blood gases, plasma creatinine, liver function tests
- Recording of any adverse events grade > 2 or adverse events of any grade (according to the CTCAE classification version 5) if the impact on medical therapy or further management is significant
- Recording of concomitant treatments (if there are any changes compared to those recorded at the pre-inclusion visit)
- Recording of the tumour assessment performed prior to the follow-up or surveillance visit
- A final blood draw of 30 ml

### Study stopping rules

The study may be suspended or discontinued by the sponsor, in consultation with the coordinator, or at the request of the competent authorities and/or the Ethics Committee (Comité de Protection des Personnes) if patient accrual is insufficient.

### Premature withdrawal from the study

The following reasons may justify premature withdrawal of a patient from the study:

- Failure to perform post-operative blood tests
- Patient not eligible for treatment with immunotherapy using an anti-PD1 monoclonal antibody or targeted therapy using BRAF and MEK inhibitors in the adjuvant setting
- Progressive disease
- Death of the patient
- Withdrawal of consent

Specific case of patients who:

- Present major toxicity requiring definitive discontinuation of adjuvant therapy
- Present toxicity requiring postponement of treatment or reduced treatment dose
- Refuse to pursue adjuvant therapy

These patients will be maintained in the study, and blood draws will continue to be performed up to 24 months after initiation of adjuvant therapy, with the patient’s consent.

Participants in the study may withdraw their consent at any time without justification, for whatever reason, and this will in no way affect their right to continue to be treated by their physician.

### Blood draws, sample preparation and transfer

Blood sampling will be sheduled for ctDNA analysis as follows:

- Before surgery (the day before or on the day of surgery before the start of surgery)
- At the post-operative visit (within 2 to 3 weeks after surgery)
- On the day of the consultation when adjuvant therapy is initiated (before the administration of adjuvant therapy)
- At each follow-up for tumour assessment (every 3 to 4 months ± 2 weeks) for the 12 months of adjuvant therapy
- At each follow-up for tumour assessment (every 6 months ± 2 weeks) for the 12 months of surveillance
- At the time of relapse

Blood samples will be drawn in Cell Free DNA collection tubes (Streck or equivalent). The 3 tubes of 10 ml of blood will be centrifuged on site in each centre for 10 min at 1,600 g at room temperature. The supernatants will be transferred to a 15 ml conical tube and centrifuged for 10 min at 6,000 g at room temperature. Around 10 to 15 ml of plasma will be retrieved in 5 ml cryotubes. ctDNA extracted from 5 ml plasma will be analysed on-site after by droplet digital PCR (ddPCR) (Váraljai R et al., 2019).

The tubes of plasma will be frozen at -80 °C for later analysis, and biobanking. The samples will be collected and transported, as per the rate of accrual in each participating centre, or at least once per year. The tubes will be transported to the laboratory for analysis on dry ice by a dedicated transporter and in compliance with current regulations.

### Tissue samples (operative tumour samples and lymph nodes)

Tissue samples (tumour samples and lymph nodes) retrieved during surgery will be analysed according to standard procedures, then transferred, as per the rate of accrual in each participating centre, or at least once per year, by a dedicated transporter and in compliance with current regulations, to the laboratory.

### DNA sequencing and digital droplet-PCR

To determine the genetic characteristics of the tumour, high-throughput sequencing will be performed on the initial tissue sample: After extraction of DNA (Qiagen, AllPrep FFPE), analysis of a 517-gene panel of interest in oncology will be performed (Agilent, SureSelect XT) with a depth of at least 1,000x (illumina, NextSeq 550). The results of tumour sequencing will enable exhaustive profiling of the initial lesions and mutations will be classified as clonal and subclonal. A clonal mutation is defined as a mutation that is common to all tumour cells, and a subclonal mutation is defined as a mutation that is present in only a subset of cells in the lesion. The selection of 3 clonal and subclonal mutations per patient will constitute a personalized profile for follow-up for each patient, specific to their tumour. For each mutation, specific, bespoken probes will be synthesized for analysis by droplet digital PCR (ddPCR, BioRad QX200).

The blood samples collected will be centrifuged as per current standards for liquid biopsy (double centrifugation at high then very high speed) and DNA will be extracted from the plasma (Qiagen, free nucleic acids kit). Extracted nucleic acids will be quantified by ddPCR (ID Solutions, quantification kit), and the fragmentation profiles will be analysed (Agilent, Fragment Analyzer). The qualification of the extracted nucleic acids will ensure the absence of any contamination by genomic DNA derived from white blood cell lysis.

All DNA samples extracted from plasma collected in the study will be analysed by ddPCR using the bespoken probes synthesized for each patient. ddPCR uses a preliminary step to denature the DNA strands, enabling detection of variants with low allele frequency (as low as 0.005%). This sensitivity will enable the detection of residual disease by the monitoring of plasma DNA concentration, and also by the follow-up of tumor clones and subclones specific to each patient (Váraljai R et al., 2019).

### Biobank collection

Any leftover or unused plasma extracted from the blood samples performed in the course of this study, or leftover or unused DNA extracted from the tissue samples, will be conserved to constitute a biobank, to enable future translational research in oncology.

The collection will be stored at the sponsor’s biobanking center for future research purposes on the same topic, namely to search for gene mutations implicated in tumour processes.

The samples and associated data may be shared with national or international research groups within the European Union.

An information leaflet and consent form will be given to each patient at the start of the study to obtain their consent for participation in the biobank, including leftover and/or unused volumes from plasma and DNA extracts from the blood draws and tissue sampling performed in the course of this study.

### Primary endpoint

The primary endpoint is the presence of ctDNA detected with 2 to 3 weeks after surgery, defined as a number of copies of mutant DNA ≥ 1 per ml of plasma, calculated on the basis of allele frequency of a detectable clonal mutation, as a fraction of the total circulating free DNA.ctDNA will be evaluated during follow-up, and is defined as follows:

- Quantitatively, by the number of mutant copies of DNA per ml of plasma.
- Qualitatively, by the presence or absence of ctDNA and clonal evolution.

The presence of ctDNA is defined as the number of mutant copies of DNA per ml of plasma. Clonal evolution will consist in the monitoring of 3 clonal and subclonal mutations identified by analysis of the initial tumor sample. Absolute and relative variations in ctDNA during follow-up will also be analysed.

### Secondary endpoints

- Recurrence-free survival (RFS) is defined as the time from inclusion to loco-regional relapse, distant metastasis or all-cause death, whichever occurs first.
- Distant metastasis-free survival is defined as the time from inclusion to distant metastasis or all-cause death, whichever occurs first.
- Melanoma specific survival is defined as the time from inclusion to cancer-related death.

## Discussion

Recent results have been reported [23], showing that liquid biopsy proved suitable for follow-up of patients with stgae III B-D, IV melanoma, receiving adjuvant immunotherapy. In this translational study, baseline ctDNA positivity in resected stage III and IV melanoma was reported to be not highly prevalent (16%) and predictive of a poorer recurrence-free survival (HR 1.87) and distant-metastasis-free survival (HR 2.86). Further, baseline ctDNA combined with other biomarkers (tumor molecular burden, IFNγ) were more predictive of recurrence than any other biomarker alone.

In the landscape of studies investigating the predictive value of ctDNA for treatment response or prognosis in melanoma [25], the present PERCIMEL study has several original features:

- Standardized follow-up at key timepoints during management with blood tests performed, at baseline, prior to surgery; at the first post-operative visit; at initiation of adjuvant therapy; at each follow-up visit up during the 12 months of adjuvant therapy; at each follow-up visit during the 12 months of surveillance; at the time of occurrence of relapse
- Personalized follow-up: to determine the genetic characteristics of the tumour, high-throughput sequencing of 517 genes known to be of interest in cancer will be performed on tissue samples from the initial lesion of each patient. The selection of 3 clonal and subclonal mutations per patient will make it possible to obtain a personalized follow-up marker for each patient, specific to each tumour. For each mutation, the use of tailor-made detection probes will enable personalized analysis by droplet digital PCR (ddPCR) during follow-up.
- Optimized technique: by partitioning samples into thousands of nanoliter-sized droplets, ddPCR enables the detection of variants with low allele frequency (0.005%), making it the most sensitive technique available today. This high sensitivity technique also enables the detection of any potential residual disease, both via monitoring of the DNA concentration in the plasma, and via the monitoring of tumoral clones and subclones specific to each patient.

PERCIMEL study is designed to provide scientific evidence that ctDNA can be used to predict the recurrence of patients with melanoma treated with adjuvant immunotherapy or kinase inhibitors, and to define the notion of molecular recurrence in resected stage III/IV melanomas.

## Data Availability

The datasets obtained during the current study, data management procedures or the full protocol will be available from the corresponding author upon reasonable request.

## Abbreviations

PD1/PDL1: Programmed cell death-1 / programmed cell death ligand-1
CTLA-4: Cytotoxic T-lymphocyte antigen-4
BRAF: B-Raf proto-oncogene
MEK: Mitogen-activated protein kinase kinase
RFS: Recurrence-Free Survival
HR: Hazard Ratio
ctDNA: Circulating Tumor DNA
LDH: Lactate Dehydrogenase
EudraCT: European Union Drug Regulating Authorities Clinical Trials
WHO: World Health Organization
ROC: Receiving Operating Characteristic
ANOVA: Analysis Of Variance
CTCAE: Common Toxicity Criteria For Adverse Events
CT: Computed Tomography
PET: Positon Emission Tomography
PCR: Polymerase Chain Reaction
ddPCR: Droplet Digital PCR
IFNγ: Interferon Gamma
GIRCI: Groupement Interrégional de Recherche clinique et d'Innovation
